# Still ‘dairy farm fever’? A Bayesian model for leptospirosis notification data in New Zealand

**DOI:** 10.1098/rsif.2020.0964

**Published:** 2021-02-17

**Authors:** Jackie Benschop, Shahista Nisa, Simon E. F. Spencer

**Affiliations:** ^1^ School of Veterinary Science, Massey University, Palmerston North, New Zealand; ^2^ Department of Statistics, University of Warwick, Coventry, UK

**Keywords:** data imputation, Markov chain Monte Carlo, epidemiology, leptospirosis

## Abstract

Routinely collected public health surveillance data are often partially complete, yet remain a useful source by which to monitor incidence and track progress during disease intervention. In the 1970s, leptospirosis in New Zealand (NZ) was known as ‘dairy farm fever’ and the disease was frequently associated with serovars Hardjo and Pomona. To reduce infection, interventions such as vaccination of dairy cattle with these two serovars was implemented. These interventions have been associated with significant reduction in leptospirosis incidence, however, livestock-based occupations continue to predominate notifications. In recent years, diagnosis is increasingly made by nucleic acid detection which currently does not provide serovar information. Serovar information can assist in linking the recognized maintenance host, such as livestock and wildlife, to infecting serovars in human cases which can feed back into the design of intervention strategies. In this study, confirmed and probable leptospirosis notification data from 1 January 1999 to 31 December 2016 were used to build a model to impute the number of cases from different occupational groups based on serovar and month of occurrence. We imputed missing occupation and serovar data within a Bayesian framework assuming a Poisson process for the occurrence of notified cases. The dataset contained 1430 notified cases, of which 927 had a specific occupation (181 dairy farmers, 45 dry stock farmers, 454 meatworkers, 247 other) while the remaining 503 had non-specified occupations. Of the 1430 cases, 1036 had specified serovars (231 Ballum, 460 Hardjo, 249 Pomona, 96 Tarassovi) while the remaining 394 had an unknown serovar. Thus, 47% (674/1430) of observations had both a serovar and a specific occupation. The results show that although all occupations have some degree of under-reporting, dry stock farmers were most strongly affected and were inferred to contribute as many cases as dairy farmers to the burden of disease, despite dairy farmer being recorded much more frequently. Rather than discard records with some missingness, we have illustrated how mathematical modelling can be used to leverage information from these partially complete cases. Our finding provides important evidence for reassessing the current minimal use of animal vaccinations in dry stock. Improving the capture of specific farming type in case report forms is an important next step.

## 1. Introduction

Leptospirosis is an important multi-host, multi-pathogen zoonosis with global annual rates of greater than 1 million cases and 60 000 deaths [1]. Humans are infected through direct or indirect contact with urine from infected mammals including wildlife, rodents, farmed species and pets [2]. The leptospirosis disease system is complex in terms of taxonomy, diagnostics, human-risk behaviours and diversity of animal hosts and infecting species thus understanding the local epidemiology is crucial to developing effective interventions.

In New Zealand, the number of incident cases have reduced significantly since the time of ‘dairy farm fever’ in the 1970s after the introduction of dairy cattle and pig vaccination programmes; however, there is still a high incidence of leptospirosis compared with other high-income countries [3]. The burden falls largely on rural communities and on Māori, the indigenous people of New Zealand [4]. From 1 January 2017 to 31 December 2019, 421 new patients were notified (average of 140 cases per annum, with incidence annualized for 2019). This represents an approximate 89% increase in annual incidence compared with the 5 years prior (2012–2016, average of 74 cases per annum) [4]. This increase coupled with a long-term change in serovar trends [4,5] and more frequent reporting of clusters of cases [6,7], point to a changing epidemiology. Despite these changes, farmers and meatworkers remain most at risk with at least 75% of notified cases from these two occupational groups. Key intervention strategies for both groups include the use of personal protective equipment (PPE) and rodent control while farmers’ strategies also include the use of livestock vaccination. New Zealand has a ‘no-fault’ worker compensation scheme administered by the Accident Compensation Corporation (ACC) which recognizes the strong link between occupation and leptospirosis. People who develop the disease resulting from their employment are eligible for cover.

An accurate knowledge of occupation is essential for uncovering the burden of disease, designing new intervention strategies and ascertaining the effectiveness of current strategies. Although notification data should contain the occupation for each case, sometimes it is recorded as ‘unknown’ and often there is no differentiation beyond farmer for different classes of farming. Within livestock farmers, notably dairy and dry stock need to be differentiated, as there are some specific risks and associated interventions. Dry stock farming encompasses the pasture grazing of beef cattle, sheep, and deer for meat, wool and velvet production. In comparison, dairy cattle are fed supplements, milked daily for 9 months of the year and have calves removed from the dams within 24 h. Thus, the dairy system relies on much more direct animal–human contact.

The aim of this model is to impute the numbers of reported cases that come from dairy farmers, dry stock farmers, meatworkers and ‘other’ occupations, based on the serovar and month of occurrence of the case.

## 2. Methods

### 2.1. Notification data

Routinely collected surveillance data were extracted from New Zealand’s notifiable disease database (EpiSurv) from 1 January 1999 to 31 December 2016. Notification data were supplemented with serovar data from the Leptospira Reference Laboratory (Institute of Environmental Science and Research). A total of 1557 cases were notified in this period. For this study, only confirmed and/or probable cases [8] that had serovars as Ballum, Hardjo, Pomona, Tarassovi or unknown were analysed (table 1). Cases classified as either under investigation or unknown (*n* = 69) and cases with serovars Copenhageni, Canicola, Australis, Grippotyphosa and Bratislava (*n* = 58) were excluded.

**Table 1.** Table summarizing number of notified leptospirosis cases by occupation and serovar.

occupation	Ballum	Hardjo	Pomona	Tarassovi	unknown	total
dairy farmer	25	56	5	51	44	181
dry stock farmer	10	11	8	0	16	45
meatworker	6	190	148	6	104	454
other	99	42	14	3	89	247
farmer	78	140	60	33	95	406
unknown	13	21	14	3	46	97
total	231	460	249	96	394	1430

All occupations reported as a dairy farmer, milker or a farmer whose animal exposures and activities as part of their employment included dairy cattle were classified as a dairy farmer. All occupations reported as dry stock farmer, pig farmer, deer farmer, beef farmer, bull farmer and sheep farmer were classified as a dry stock farmer. All occupations reported as butcher, abattoir worker, freezing worker, meatworker or as any position at an abattoir that includes the handling of animals were classified as a meatworker. All occupations reported as farmer whose activities as part of their employment could not distinguish the type of animals being farmed were classified as farmer. All occupations that were neither farmer nor meatworker were categorized as ‘other’ including retired, unemployed and unknown occupation. A total of 1430 cases were available for analysis. Serovar data were available for 1036 cases. The month of the cases was the month the cases were notified to the Ministry of Health.

### 2.2. Notation

Let *λ*_*i*,*y*_ represent the average rate of notified cases from occupation *i* ∈ *I* = {dairy farmer, dry stock farmer, meatworker, other} in year *y* ∈ *Y* = {1999, …, 2016}. Let *μ*_*i*,*m*_ denote the proportion of notified cases from occupation *i* ∈ *I* that occur in calendar month *m* ∈ *M* = {Jan, …, Dec}. Let *p*_*i*,*j*_ be the proportion of notified cases from occupation *i* ∈ *I* that are of serovar *j* ∈ *J* = {Ballum, Hardjo, Pomona, Tarassovi}. Let *θ* denote the probability that a serovar is recorded, given that the case has been notified. Let *ϕ*_*k*,*i*_ be the probability that a case with occupation *i* ∈ *I* is recorded as having occupation $k\in I^{+}=I\cup \{\;\mathrm{farmer},\mathrm{unknown}\}$, where *I*^+^ is the complete set of possible recorded occupations including farmer and unknown. Let *n*_*i*,*y*_ be the number of individuals in New Zealand with occupation *i* ∈ *I* in year *y* ∈ *Y*. Finally, let *X*_*i*,*j*,*t*_ denote the number of recorded cases with occupation *i* ∈ *I*^+^ and serovar $j\in J^{+}=J\cup \{\mathrm{unknown}\}$ that occur in study month *t* ∈ *M* × *Y*. Denominator information by occupation was obtained from the New Zealand census, given in table 2. A linear trend over time was fitted to the log of the populations to interpolate between years, leading to exponential growth or decay in each occupation.

**Table 2.** Population sizes obtained from the New Zealand census.

year	dairy farmer	dry stock farmer	meatworker	other
2001	26 331	46 983	20 157	3 727 278
2006	24 795	46 194	18 858	3 938 100
2013	26 577	44 976	14 997	4 155 498

### 2.3. Model

We assume that notified cases occur from a Poisson process, so that for *i* ∈ *I*, *j* ∈ *J* and *t* ∈ *M* × *Y*, *X*_*i*,*j*,*t*_ ∼ Pois(*ϕ*_*i*,*i*_*n*_*i*,*y*(*t*)_*λ*_*i*,*y*(*t*)_*μ*_*i*,*m*(*t*)_*p*_*i*,*j*_*θ*), where *y*(*t*) is the year of study month *t* and *m*(*t*) is the calendar month of study month *t*. Similarly, for cases with serovar *j* = unknown, we assume that *X*_*i*,*j*,*t*_ ∼ Pois(*ϕ*_*i*,*i*_*n*_*i*,*y*(*t*)_*λ*_*i*,*y*(*t*)_*μ*_*i*,*m*(*t*)_(1 − *θ*)), for *i* ∈ *I* and *t* ∈ *M* × *Y*.

For the non-specific occupations *k* ∈ {farmer, unknown}, *j* ∈ *J* and *t* ∈ *M* × *Y*, we assume

$$X_{k,j,t}∼\mathrm{Pois}\left(\sum_{i\in I}\phi_{k,i}n_{i,y(t)}\lambda_{i,y(t)}\mu_{i,m(t)}p_{i,j}\theta \right),$$

and finally for the non-specific occupations that have *j* = unknown,

$$X_{k,j,t}∼\mathrm{Pois}\left(\sum_{i\in I}\phi_{k,i}n_{i,y(t)}\lambda_{i,y(t)}\mu_{i,m(t)}(1-\theta )\right).$$

The model expectations can be summarized compactly in matrix form as

$$E[X_{t}]=\Phi D_{t}P\Theta ,$$

where $X_{t}={\left[X_{i,j,t}\right]}_{i\in I^{+},j\in J}$; $\Phi ={\left[\phi_{k,i}\right]}_{k\in I^{+},i\in I}$, noting that *ϕ*_*k*,*i*_ = 0 for *i*, *k* ∈ *I* with *i* ≠ *k*; ***D***_*t*_ = diag[(*n*_*i*,*y*(*t*)_*λ*_*i*,*y*(*t*)_*μ*_*i*,*m*(*t*)_)_*i*∈*I*_]; ***P*** = [*p*_*i*,*j*_]_*i*∈*I*,*j*∈*J*_ and

$$\Theta =\left[\begin{array}{ccccc} \theta & 0 & 0 & 0 & 1-\theta \\ 0 & \theta & 0 & 0 & 1-\theta \\ 0 & 0 & \theta & 0 & 1-\theta \\ 0 & 0 & 0 & \theta & 1-\theta \end{array}\right].$$

### 2.4. Priors

Priors for probability vectors were chosen to be Dirichlet distributions to ensure that they summed to 1, and the infection rates were chosen to follow the gamma distribution. For *i* ∈ *I* and *y* ∈ *Y*:

$$\begin{array}{rl} \lambda_{i,y} & ∼\mathrm{gamma}(1,1)\\ {\left(\mu_{i,m}\right)}_{m\in M} & ∼\mathrm{Dirichlet}(\alpha_{\mu })\\ {\left(\phi_{k,i}\right)}_{k\in I^{+}} & ∼\mathrm{Dirichlet}(\alpha_{\phi ,i})\\ {\left(p_{i,j}\right)}_{\;j\in J} & ∼\mathrm{Dirichlet}(\alpha_{\;p,i})\\ \theta & ∼\mathrm{beta}(0.5,0.5). \end{array}$$

We chose to prevent specific occupations from being recorded as each other by putting zeros in ***α***_*ϕ*,*i*_ for the corresponding entries. This prevents a dairy farmer from being recorded as a meatworker (or vice versa) while allowing all occupations to be recorded either correctly or as farmer or unknown. We take ***α***_*ϕ*,*i*_ to be 0.8/3 in the non-zero entries, following the non-informative prior recommended by Berger *et al.* [9] of using 0.8 divided by the dimension.

We assume ***α***_*p*,*i*_ = (0.2, …, 0.2) also following Berger *et al.* [9] and $\alpha_{\mu }=(0.5,\ldots ,0.5)$, which is the Jeffreys prior.

### 2.5. MCMC algorithm

The prior distributions for *λ*_*i*,*y*_ and *p*_*i*,*j*_ are conjugate with respect to the data from occupation *i*, but non-conjugate for the non-specific occupations. This suggests that the parameters’ conditional distributions on the conjugate part of the data might be good Metropolis–Hastings proposals that can be accepted or rejected based on the likelihood for remainder of the data.

For *μ*_*i*,*m*_ and *ϕ*_*k*,*i*_, an adaptive Dirichlet random walk scheme was used to target an acceptance rate of approximately 25%. The scheme uses a mixture of the conjugate proposal described above and the current location of the chain. The mixing parameter *β* is adjusted automatically during the burn-in to produce the desired acceptance rate via the following rules. If a proposal is accepted then β↦max{0,β−3} and if a proposal is rejected then β↦β+1. These rules concentrate the proposal around the current location if the acceptance rate is too low.

The corresponding Metropolis–Hastings proposals were:

$$\begin{array}{rl} \lambda_{i,y}^{\prime } & ∼\mathrm{gamma}\left(1+\sum_{t:y(t)=y}\sum_{\;j\in J^{+}}X_{i,j,t},1+n_{i,y(t)}\phi_{i,i}\right)\\ {\left(p_{i,j}^{\prime }\right)}_{\;j\in J} & ∼\mathrm{Dirichlet}\left(\alpha_{\;p,i}+{\left(\sum_{t\in M\times Y}X_{i,j,t}\right)}_{\;j\in J}\right)\\ {\left(\phi_{k,i}^{\prime }\right)}_{k\in I^{+}} & ∼\mathrm{Dirichlet}(\alpha_{\phi ,i}+\beta_{\phi ,i}{\left(\phi_{k,i}\right)}_{k\in I^{+}})\\ {\left(\mu_{i,m}^{\prime }\right)}_{m\in M} & ∼\mathrm{Dirichlet}(\alpha_{\mu ,i}+\beta_{\mu ,i}{\left(\mu_{i,m}\right)}_{m\in M}). \end{array}$$

Finally, the parameter *θ* has a conjugate prior and can be sampled from its full conditional distribution,

$$\theta |X∼\mathrm{beta}\left(0.5+\sum_{i\in I^{+}}\sum_{\;j\in J}\sum_{t\in M\times Y}X_{i,j,t},0.5+\sum_{i\in I^{+}}\sum_{t\in M\times Y}X_{i,\mathrm{unknown},t}\right).$$

This MCMC algorithm was implemented in R [10] and run from random starting locations for 25 000 iterations with a burn-in of 5000 iterations and a thinning of 10. Convergence was assessed by comparing multiple independent chains and visual inspection of trace plots.

## 3. Results

**Figure 1.**
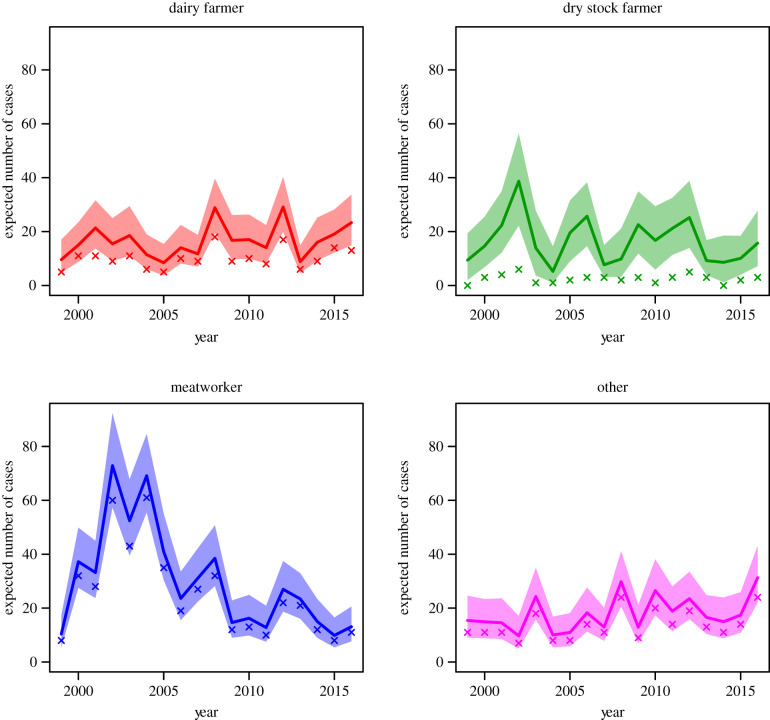
Posterior median and 90% CI for the expected number of notified leptospirosis cases from each occupation in each year. Crosses indicate the number of cases reported for each occupation.

Figure 1 shows the imputed contribution of each occupation to the burden of leptospirosis notifications through time (years). The crosses indicate the reported number of notified leptospirosis cases for each occupation. Although all occupations show some degree of under-reporting (due to occupations being recorded as farmer or unknown), dry stock farmers are most strongly affected. Interestingly, dry stock farmers were inferred to contribute as many cases as dairy farmers to the burden of disease, despite dairy farmer being recorded much more frequently as the occupation for notified cases. For comparison, posterior median values of annual incidence per 100 000 people of each occupation are provided in table 3. These show that meatworkers generally have the highest incidence rate followed by dairy and then dry stock farmers.

**Table 3.** Posterior median estimates of the number of notified leptospirosis cases per year per 100 000 people of each occupation.

	dairy farmer	dry stock farmer	meatworker	other
1999	37.47	19.86	47.90	0.42
2000	58.55	31.04	176.21	0.40
2001	83.14	47.52	161.18	0.39
2002	60.02	82.59	362.80	0.26
2003	71.96	29.08	267.64	0.64
2004	44.36	11.26	361.86	0.26
2005	32.68	42.16	219.79	0.28
2006	54.19	55.67	129.99	0.47
2007	45.34	16.07	175.59	0.33
2008	111.17	21.46	222.75	0.75
2009	64.44	49.42	86.95	0.32
2010	65.49	36.78	98.81	0.65
2011	53.93	46.74	79.86	0.46
2012	111.70	55.81	172.67	0.57
2013	33.90	20.50	153.78	0.40
2014	61.29	19.14	101.15	0.36
2015	72.93	22.54	68.14	0.41
2016	88.98	35.32	92.76	0.73

**Figure 2.**
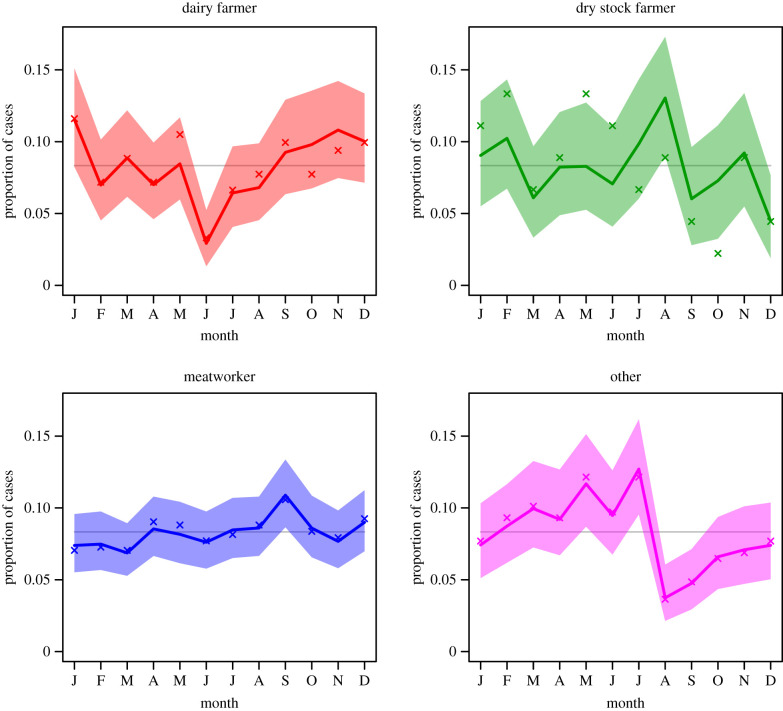
Posterior median and 90% CI for the proportion of notified leptospirosis cases from each month by occupation. Crosses indicate the proportions in the observed data. Horizontal line shows equal proportions.

Figure 2 shows the posterior distribution of the proportion of notified leptospirosis cases to occur in each month of the year, by occupation. The crosses indicate the proportions observed in the corresponding data. There are striking falls in both predicted and observed proportions in dairy farmers in the early winter and in other occupations in the early spring. These coincide with the start of the dry period, when cows are not milked, for dairy farmers and as temperatures start to rise when rodents leave the built environment for other occupations.

**Figure 3.**
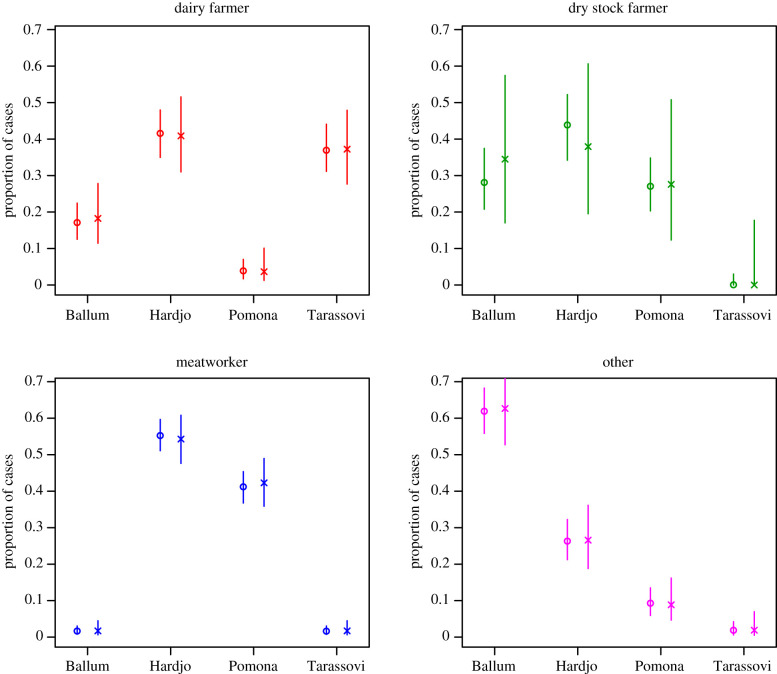
Posterior median and 90% CI for the proportion of notified leptospirosis cases from each serovar by occupation (circles). Crosses indicate the proportions in the observed data with 90% Goodman multinomial CIs.

The posterior median and 90% CI for the proportion of notified leptospirosis cases from each serovar by occupation is shown in figure 3 (circles). The intervals alongside (crosses) indicate the corresponding observation in the dataset, with 90% Goodman multinomial confidence intervals [11]. Although the inferred distributions follow the observed data closely, there is a reduction in the uncertainty due to the additional cases recorded as farmer or unknown.

**Figure 4.**
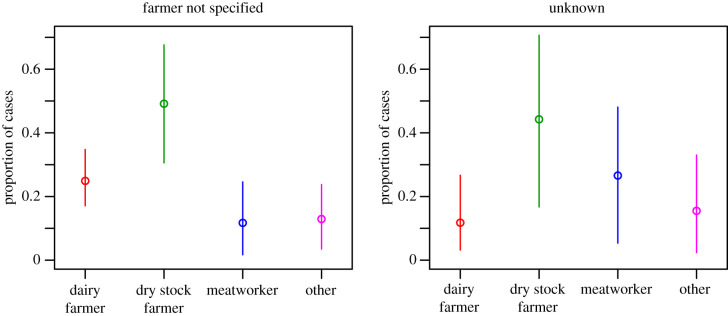
Posterior median and 90% CI for the *inferred* occupation of notified leptospirosis cases *recorded* as farmer (left plot) or unknown (right plot).

**Figure 5.**
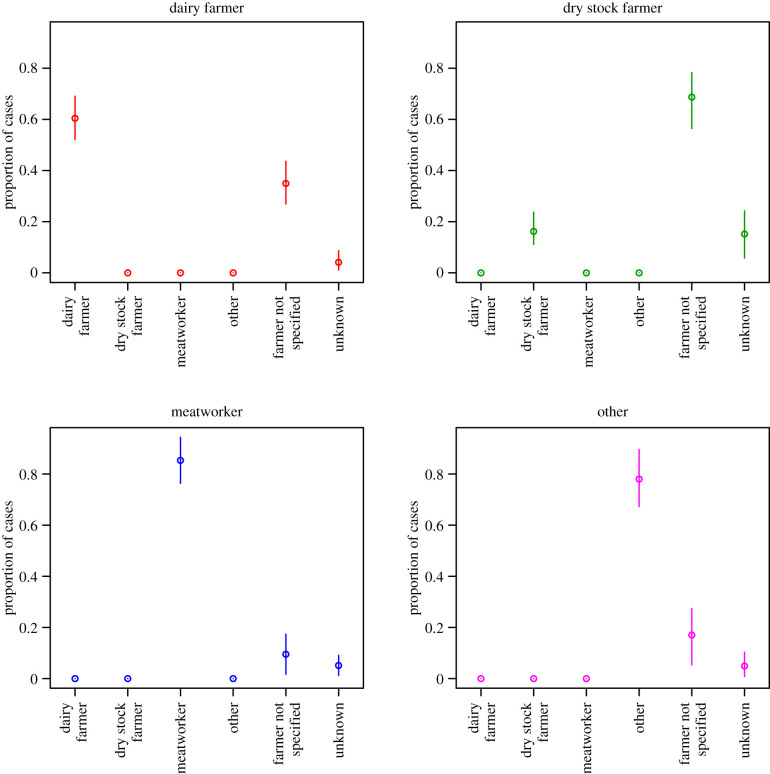
Posterior median and 90% CI for the proportion of notified leptospirosis cases with each *recorded* occupation, split by *inferred* occupation in the four panels. This presentation is the reverse of figure 4.

Figure 4 shows the inferred occupation of notified leptospirosis cases that were recorded as farmer or unknown. Unsurprisingly, a large proportion of the cases recorded as farmer were inferred to be dry stock farmers, and a smaller proportion dairy farmers. The unknown cases were more equally distributed, with dry stock farmers and meatworkers forming the largest proportions. The inverse of these proportions—the proportion of each occupation that notified correctly, as farmer or as unknown—are given in figure 5.

**Figure 6.**
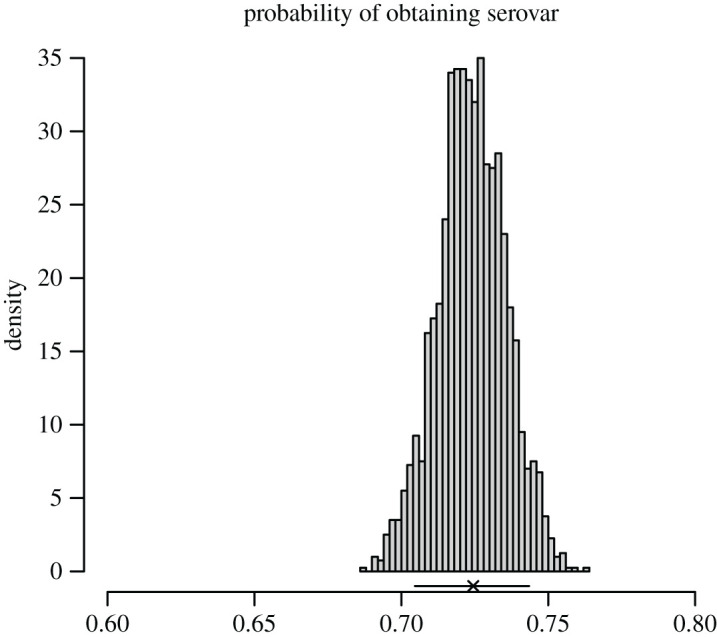
Posterior histogram for the probability that a notified leptospirosis case has a serovar recorded. Cross and horizontal lines indicate the proportion observed in the data and 90% binomial CI.

Finally, figure 6 shows the posterior distribution of the probability that a serovar is recorded for a given notified leptospirosis case. At the bottom of the figure is shown a 90% binomial confidence interval, indicating that the posterior from the model simply reflects the uncertainty in the data.

## 4. Discussion

Routinely collected notification datasets, such as this one, inevitably contain substantial amounts of missing or partially complete data. In our data, only 47% (674/1430) observations had both a serovar and a specific occupation. Rather than discard records with some missingness, we have illustrated how mathematical modelling can be used to leverage information from these partially complete cases. In particular, we inferred trends in occupational infection from the distribution of serovars by occupation, and the seasonal variations in reporting month by occupation. This additional information allowed us to attribute cases with partially complete data and subsequently make meaningful comparisons between the contributions of dairy and dry stock farmers to the burden of disease in New Zealand.

Our main finding was that dry stock farmers contributed approximately equal numbers of cases of leptospirosis as dairy farmers, however, dairy farmer was a more frequently recorded class of occupation (figure 1). Thus, this model suggests that when leptospirosis patients are interviewed to collect surveillance data, and they identify their occupation as ‘farmer’, there is a need to more finely differentiate their type of farming. This detailed occupational data collection is important as it has implications for leptospirosis prevention and control strategies. Currently, approximately 99% of dairy farms in New Zealand have a leptospirosis vaccination programme for cattle against Hardjo and Pomona [12]. However, dry stock vaccination rates are much lower, i.e. beef cattle herds (18–25%), deer herds (5–9%) and sheep flocks (less than 1%) [13]. Although dry stock farmers do not have daily contact with their animals they are exposed by other pathways. For example, a survey of 178 dry stock farmers in 2013 reported that farmers who had assisted with calving cattle and deer had seven times the risk of being seropositive for *Leptospira* [13]. Poor vaccination uptake in dry stock farming, inadvertently exposes meatworkers to risk, as they come into direct contact through yarding, slaughtering and eviscerating a large number of unvaccinated animals. Meatworkers have the highest occupation-specific incidence of leptospirosis in New Zealand (table 3). Monthly data show that a dry stock farmer infection peak in August (figure 2) which is likely due to increased animal contact at spring lambing and calving. The meatworker peak in September may be associated with the return of workers to begin processing the new seasons lambs after winter shut-down [14]. In addition, meatworkers are most often notified with the two serovars most frequently contained in livestock vaccines, i.e. Hardjo and Pomona (figure 3). Meatworkers have no agency in relation to the vaccination status of stock they process.

Interestingly, although livestock vaccines contain Hardjo and Pomona, the proportion of dairy farmers notified with leptospirosis due to Hardjo is much higher than the proportion notified with Pomona (figure 3). Hardjo antibody titres in patients may be due to another endemic serovar, Balcanica. Balcanica and Hardjo are both in the Sejroë serogroup, and thus serologically indistinguishable using current diagnostic methods [15]. In New Zealand, serovar Balcanica is maintained in the common brushtail possum (*Trichosurus vulpecula*) and does spill-over to cattle and other animals [16].

Our study identifies that dairy farmers are the occupation most commonly notified with Tarassovi (table 1). This finding is supported by a survey of 4000 dairy cattle in 200 herds performed in 2016 which showed a strong association between urinary shedding of *Leptospira* and serological titres for Tarassovi suggesting dairy cattle play an important role in the epidemiology of this serovar [12]. We further identified serovar Ballum is strongly associated with the ‘other’ occupational group (figure 3). Dairy and dry stock farmers are also notified with Ballum, however not meatworkers. In New Zealand, serovar Ballum is maintained in rodents, predominately mice (*Mus musculus*) and does spill-over to cattle [17]. It is not clear why meatworkers are less frequently notified with Ballum, but it is possible that farmers are infected through animal feed and pasture contamination.

Our results rest on the appropriateness of the assumptions in the model and this presents some limitations. For example, we assumed that all notified cases had equal probability of producing one of the serovars in the database, irrespective of time of year, location or occupation. However, there is heterogeneity in both the use of diagnostic tests for leptospirosis and in the occupations of notified cases across New Zealand [4]. The choice of which of the two tests (MAT and PCR) [18] to use is driven by variation in clinician preference. The MAT test provides serovar data but the PCR test does not. In addition, there are regional differences in occupations, e.g. dairying occupations predominate among notified leptospirosis cases in the Northland region while, in the east coast of the North Island (the Hawke’s Bay Region) meat working occupations predominate. Furthermore, these occupations themselves are associated with different serovars. The overall effect this heterogeneity has on our study results is difficult to predict. However, since a positive laboratory test is required before notification, all notified cases must have produced a blood sample for diagnosis, therefore an access to healthcare bias due to rurality would not apply to this aspect of the model.

We also assumed that the distribution of serovars for each occupation remained constant throughout the study period (1999–2016). Over this period, there have been substantial changes in New Zealand agriculture, including reduction in the meat working population associated with a 44% decrease in sheep numbers from 1994 to 2017 [19]. Additionally, there has been an approximate twofold increase in dairy production since the 1990s associated with an increase in dairy herd sizes and a resultant rise in employment within dairy sectors [20,21]. As a result, it is likely that infection pressures within dairy herds and the number of people being exposed to *Leptospira* in a dairy setting has increased. Although changes in incidence are captured by the model, such large changes indicate changing farming practices that may have affected the relative prevalence of serovars for these occupations.

This work provides important evidence for reassessing the current minimal use of leptospirosis vaccinations in dry stock. Vaccination programmes applied to dry stock should help reduce the high proportion of meatworkers and dry stock farmers being infected with the serovars in the vaccines. The effectiveness of such a vaccination programme has been demonstrated to reduce the incidence of ‘dairy farm fever’ in dairy workers in the past 40 years.

## Data accessibility

Data and code are available at SEFS Github page: https://github.com/drsimonspencer/Lepto-Missing-Data.
